# High-flow Nasal Cannula: Mechanisms of Action and Adult and Pediatric Indications

**DOI:** 10.7759/cureus.3639

**Published:** 2018-11-26

**Authors:** Frank J Lodeserto, Thomas M Lettich, Salim R Rezaie

**Affiliations:** 1 Internal Medicine, Geisinger Medical Center, Danville, USA; 2 Emergency Medicine, Methodist Hospital, San Antonio, USA

**Keywords:** respiratory failure, dead space washout, oxygen dilution, high-flow nasal cannula

## Abstract

The use of the heated and humidified high-flow nasal cannula has become increasingly popular in the treatment of patients with respiratory failure through all age groups. This article will examine the main mechanisms of actions attributed to the use of the high-flow nasal cannula and review the indications in adult and pediatric populations (outside of the neonatal period). It is unclear which of the mechanisms of action is the most important, but it may depend on the cause of the patient’s respiratory failure. This article describes the mechanism of action in an easy to remember mnemonic (HIFLOW); Heated and humidified, meets Inspiratory demands, increases Functional residual capacity (FRC), Lighter, minimizes Oxygen dilution, and Washout of pharyngeal dead space. We will also examine some of the main indications for its use in both the adult and pediatric age groups. The data for the use of high-flow nasal cannula is growing, and currently, some of the main adult indications include hypoxemic respiratory failure due to pneumonia, post-extubation, pre-oxygenation prior to intubation, acute pulmonary edema, and use in patients who are "do not resuscitate or intubate". The main pediatric indication is in infants with bronchiolitis, but other indications are being studied, such as its use in asthma, croup, pneumonia, transport of a critically ill child, and post-extubation.

## Introduction and background

The heat and humidified high-flow nasal cannula or, as most call it, high-flow nasal cannula (HFNC), isn’t just a standard nasal cannula turned up to very high flow rates. It takes gas, is able to heat it to 37^ o^C with a 100% relative humidity, and can deliver 0.21 - 1.00% fraction of inspired oxygen (FiO_2_) at flow rates of up to 60 liters (L)/min. The flow rate and FiO_2_ can be independently titrated based on a patient’s flow and FiO_2_ requirements.

There are two main companies who manufacture these devices: Vapotherm® (Exeter, NH), which has a device that can deliver flow rates of up to 50 L/min flow rates, and Fisher and Paykel Healthcare, Inc. (Auckland, New Zealand), who offers both the Optiflow™, as well as the AIRVO™ 2 device, both of which can deliver flow rates of up to 60 L/min.

Each company offers smaller cannula sizes for premature neonates, as well as children of various ages, to adult-sized cannulas. The nasal prongs should fit snuggly in the patient’s nares in order to prevent entrainment of room air around the cannula, a problem which occurs in standard nasal cannulas. Each manufacturer has a maximum flow rate for each cannula size corresponding to the size and age of the patient.

## Review

Mechanisms of actions

There are many beneficial mechanisms of action that have been attributed to the effectiveness of high-flow nasal cannula in adult and pediatric patients with respiratory failure. It is not clear which of the benefits are most important, and it may depend on the individual patient's etiology of respiratory failure. The mechanisms of action below are not listed in the order of importance but rather to give the reader an easy to remember mnemonic (HIFLOW) for the mechanisms that have been attributed to the use of high-flow nasal oxygen.

Heated and Humidified

Heated and humidified oxygen has a number of benefits compared to standard oxygen therapy. Standard oxygen therapy delivered through a nasal cannula or another device, such as a non-rebreather mask (NRBM), delivers cold (not warmed) and dry (not humidified) gas. This cold, dry gas can lead to airway inflammation, increase airway resistance, and impair mucociliary function, possibly impairing secretion clearance [1]. Also, a significant amount of energy is expended by individuals to both warm and humidify gas during normal breathing [2]. Thus, heated and humidified oxygen may improve secretion clearance, decrease airway inflammation, and also decrease energy expenditure, particularly in the setting of acute respiratory failure [1-2]. 

Inspiratory Demands

One obvious benefit is that the high-flow nasal cannula can deliver very high flow rates of gas in an attempt to match a patient's inspiratory flow demands. This is important as patients in acute respiratory failure can become extremely tachypneic, and their peak inspiratory flows (PIF), which may normally be 30 L/min - 60 L/min at rest, can reach upwards of 120 L/min in acute respiratory failure [3]. If these patients with respiratory failure (with PIF rates of up to 60 - 120 L/min and high minute volumes (> 20 L/min in some adults)) are placed on a 15 L/min NRB mask, then this may not provide adequate support. This will be discussed later in this review when we discuss the concept of oxygen dilution. One of the main mechanisms to improve a patient's work of breathing is to attempt to match their peak inspiratory flow demands with the use of a high-flow device.

Functional Residual Capacity

There is some debate over the level of positive end-expiratory pressure (PEEP) provided by high-flow devices. Best estimates are 1 cm H_2_0 of PEEP for every 10 L/min of flow delivered with closed mouth breathing [4-5].

There has been a lot of variation in studies measuring how much PEEP that high-flow cannulas can generate. This may vary from patient to patient as there are many factors that can affect how much PEEP can actually be delivered to a patient. Factors, such as the patient’s size (obese, adult, child), the liter flow rate being delivered (L/min), and mouth open versus mouth closed breathing (pressure may escape when a patient's mouth is open), can all affect the amount of PEEP being delivered [4].

The debate can continue, but it appears that HFNC can increase a patient’s functional residual capacity (FRC) or the lung volume at the end of expiration, which is something that PEEP usually improves. A study by Riera et al. showed the use of HFNC increased end-expiratory lung impedance (EELI), implying there was an improvement in FRC [6]. They used electrical impedance tomography (EIT), a noninvasive, real-time imaging method that provides a cross-sectional ventilation image of the lung, to demonstrate an increased EELI.

It also appears that the use of HFNC can decrease preload by increasing intrathoracic pressure, again another feature commonly attributed to the addition of PEEP. Roca et al. demonstrated in a sequential interval study on 10 patients (New York Heart Association (NYHA) Classification III - heart failure but not in an acute congestive heart failure (CHF) exacerbation) that the use of HIFLOW caused an inspiratory collapse of the inferior vena cava (IVC) from the patient's baseline which was measured by echocardiogram [7].

High-flow nasal cannula use seems to cause alveolar recruitment and increased FRC, as well as increased intrathoracic pressure, likely as a result of the added PEEP; however, it is not certain if perhaps another mechanism may be responsible for these findings.

Lighter

Patients often prefer the use of HFNC to that of non-invasive continuous or bilevel positive pressure ventilation (CPAP or BPAP) because the tight-fitting mask can be uncomfortable for some patients. They may even prefer it to the standard nasal cannula (NC) because of the warmed, humidified gases that won’t dry their mucosa like standard oxygen therapy [5]. This may lead to higher compliance with HFNC and perhaps an improvement in the patient’s oxygenation and work of breathing.

O_2_ Dilution

It is taught that 1 L/min administered via nasal cannula will deliver ~4% FiO_2_ above room air (21%). Thus, 1 L/min via the NC should deliver ~ 25% FiO_2_, while 2 L/min should deliver 29% FiO_2_ (Table 1). Many refer to this as the “1:4 rule”, and this concept that is taught widely; let's examine this more closely. 

**Table 1:** "4:1" Rule FiO_2_: fraction of inspired oxygen

Liter Flow	FiO_2_
1	25%
2	29%
3	33%
4	37%

**Figure 1:**
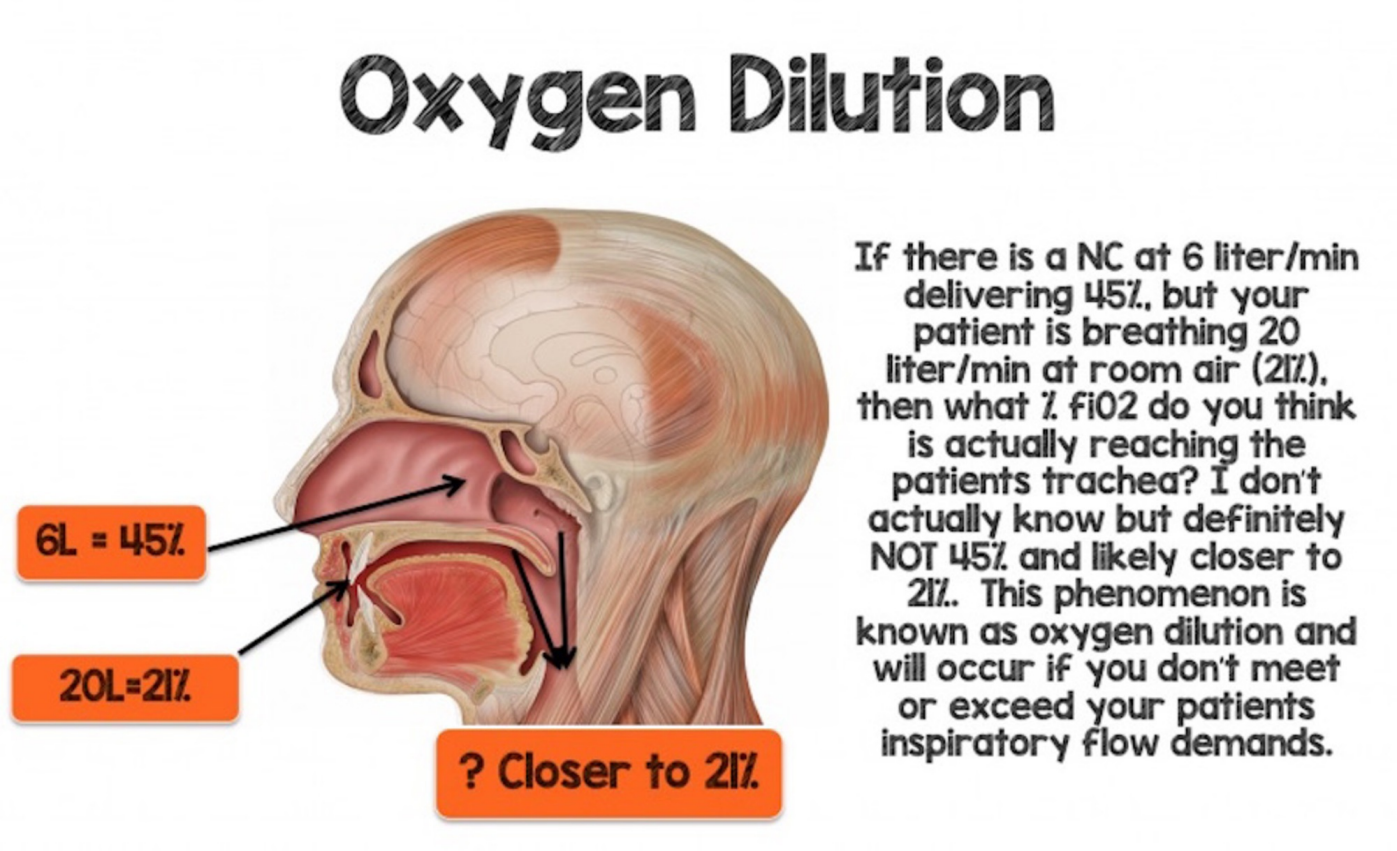
Oxygen Dilution FiO_2_: fraction of inspired oxygen; L: liter; NC: nasal cannula Image courtesy of www.rebelem.com

Consider a 70 kg male patient breathing 30 - 40 beats per minute (bpm) with normal tidal volumes (~500 mL’s) who develops acute hypoxemia. This patient’s minute ventilation would be between 15 - 20 L/min. If this patient is placed on 6 L/min NC, this should theoretically deliver an FiO_2_ ~ 45% (6 L x 4% = 24 + room air (21%) = 45%) if the “1:4 rule” holds true. If this patient is breathing 15 - 20 L through his mouth and nares (around the nasal cannula) at 21%, then the gas reaching the patient's trachea will be diluted with room air and be closer to 21% FiO_2_ rather than 45% FiO_2_ (Figure 1).

**Figure 2:**
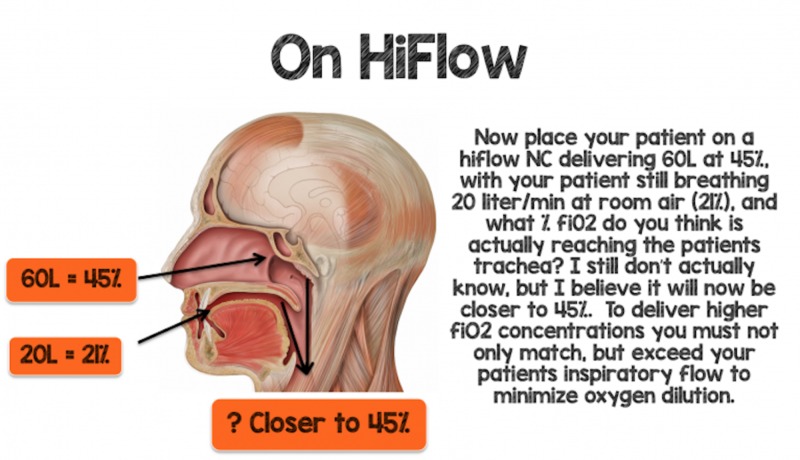
Minimizing Oxygen Dilution FiO_2_: fraction of inspired oxygen; L: liter; NC: nasal cannula Image courtesy www.rebelem.com

To deliver higher amounts of FiO_2_ effectively to a patient, the patient's minute ventilation and inspiratory demands need to be not only matched but to exceed to minimize the effects of oxygen dilution (Figure 2).

Washout of Dead-space

We can normally rebreathe a third of our previously expired tidal volume, and instead of breathing 21% (room air) and negligible amounts of carbon dioxide, we may rebreathe more like 15 - 16% oxygen and 5 - 6% carbon dioxide. This is because the previously exhaled breath (low in oxygen and containing carbon dioxide) is not fully exhaled and remains in the upper airway. When the patient takes their next breath from atmospheric gas, not all of that gas will actually enter the alveoli. In fact, it's a mixture of the new atmospheric gas (21% FiO_2_, negligible CO_2_) and their previously exhaled gas (< 21% oxygen with a larger amount of CO_2_) that enters the alveoli for gas exchange. In patients with acute respiratory failure, the percentage of gas we rebreathe gets larger, and as a result, we can rebreathe larger amounts of carbon dioxide as we inspire from a mixed reservoir from our upper airway. 

One of the major benefits of HFNC (some argue it’s actually the main benefit) is that it gives you a continuous flow of fresh gas at high-flow rates replacing or washing out the patient’s pharyngeal dead-space (the old gas low in oxygen and high in CO_2_). Each breath that the patient now re-breathes with high-flow nasal cannula will have had its carbon dioxide washed out and replaced with oxygen-rich gas and thus improving breathing efficiency [8]. 

**Figure 3:**
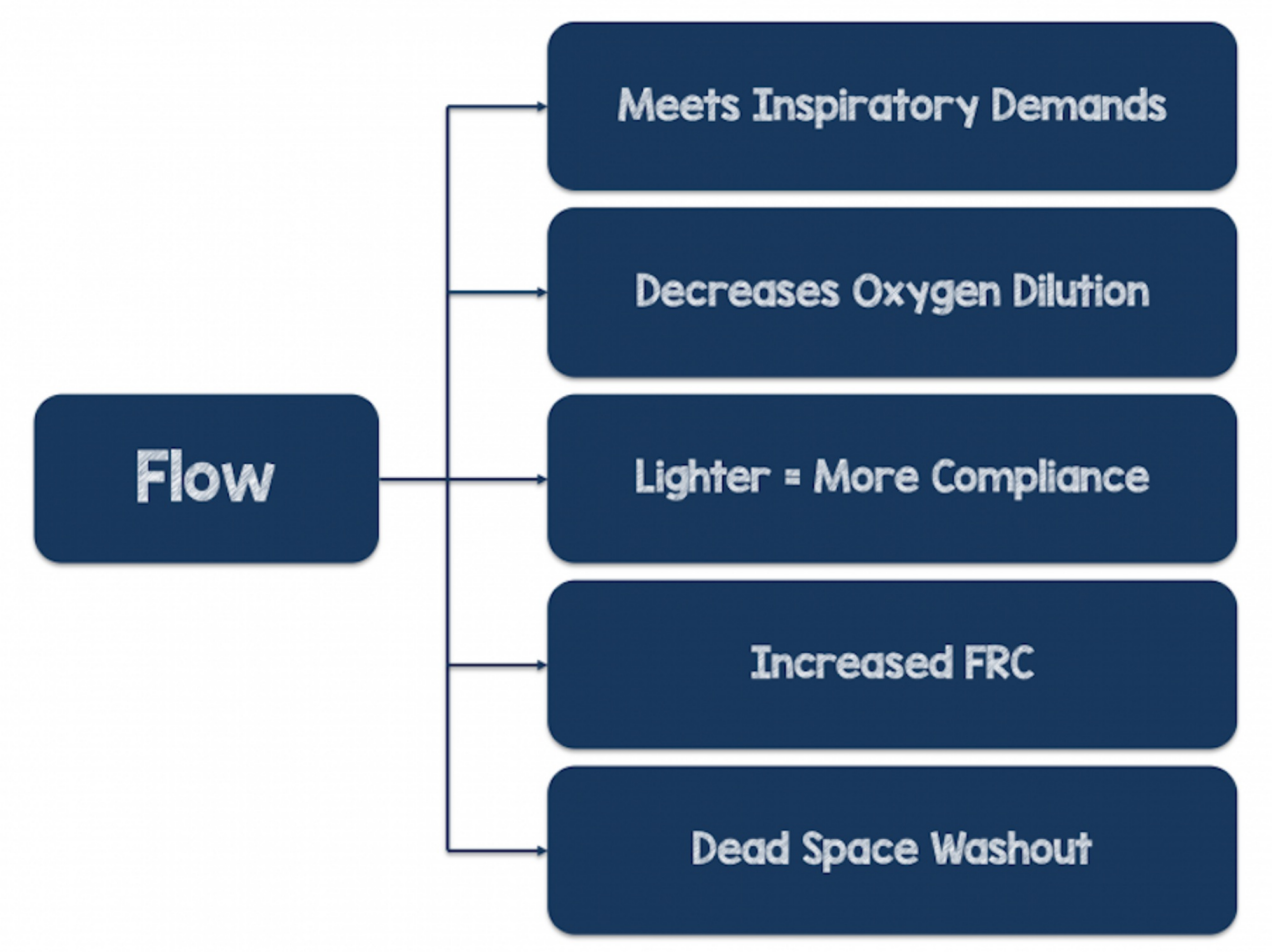
Mechanisms of Action of Flow FRC: functional residual capacity Image courtesy of www.rebelem.com

Figure 3 (below) gives you an easy to remember mnemonic to recall the mechanisms of action for the high-flow nasal cannula.

**Figure 4:**
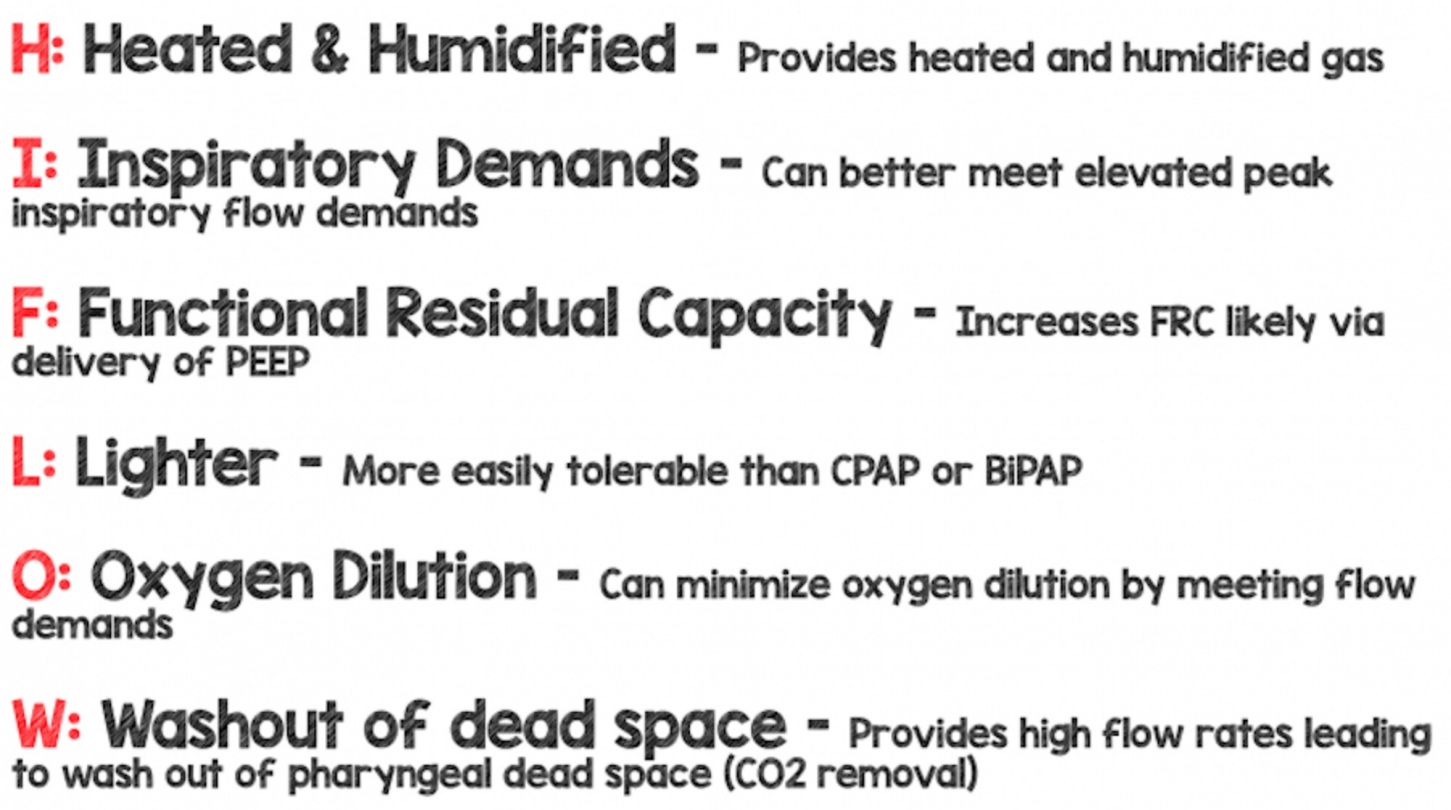
Mechanisms of Action of High-Flow Nasal Cannula BiPAP: bilevel positive airway pressure; CO_2_: carbon dioxide; CPAP: continuous positive airway pressure; FRC: functional residual capacity; PEEP: positive end-expiratory pressure Image courtesy of www.rebelem.com

The majority of the benefits of the high-flow nasal cannula, as we have discussed above, come as a result of the high flow rates that can be delivered (Figure 4). Delivering heated and humidified oxygen has significant benefits, but to optimize the effectiveness of high-flow nasal cannula for the patient, ensure that the inspiratory flow is optimized. As we will discuss in our next section, pediatric data (particularly in bronchiolitis) shows that flow rates of 2 L/kg/min are effective and well tolerated up to maximum flow rates of 60 L/kg/min in adults.

Adult indications

Acute Hypoxemic Respiratory Failure (Mainly from Community-acquired Pneumonia)

Community-acquired pneumonia would seem like an ideal indication for the use of the high-flow nasal cannula. The heated, humidified oxygen should allow improved mobilization of secretions, and its ability to minimize oxygen dilution, meet inspiratory demands, and improve end-expiratory lung volumes all seem ideal in patients with pneumonia. HFNC may allow patients to cough, mobilize secretions, and be suctioned, if necessary, all benefits that are often difficult to achieve when a patient is on noninvasive positive pressure ventilation (NIPPV). The only exception would be in patients with acute chronic obstructive pulmonary disease (COPD) exacerbations from pneumonia, as there is evidence demonstrating a decreased mortality and need for intubation with the use of NIPPV [9}.

Frat et al. conducted a multi-centered, open-label, randomized control trial with 300 patients, mainly with community-acquired pneumonia (CAP), randomized to either oxygen (non-rebreather mask), HFNC (for two days), or NIPPV (for at least 8 hrs/day for two days) [10]. The primary outcome was intubation rates, and the data did not show a statistically significant difference between the groups. Though this study seemed like a negative trial for the use of a high-flow nasal cannula, a secondary outcome (90-day all-cause mortality) showed that the use of HFNC, even after adjusting for illness severity (Acute Physiology and Chronic Health Evaluation (APACHE) II and cardiac insufficiency), was improved with the use of HFNC compared to NIPPV and standard oxygen therapy. A post hoc analysis also showed a statistically significant reduction in the intubation rates in patients with severe respiratory failure (PaO_2_/FiO_2_ ≤ 200) using HFNC. A meta-analysis by Ni et al. also demonstrated that the use of HFNC compared to NIPPV and conventional oxygen therapy (COT) was associated with a reduction in endotracheal intubation rates in acute respiratory failure [11]. 

A recent study by Azoulay et al. comparing the use of the high-flow nasal cannula to standard oxygen therapy in immunocompromised patients with acute hypoxemic respiratory failure failed to show a difference in 28-day mortality between the groups [12}. Despite improvements in oxygenation (higher Pa0_2_/Fi0_2_ ratio compared to standard oxygen therapy), high flow nasal cannula also failed to demonstrate a difference in rates of intubation, intensive care unit (ICU) length of stay, ICU-acquired infections, hospital length of stay or patient comfort and dyspnea scores.

Post-extubation (Patients at Low Risk for Reintubation)

Another unique use for high-flow nasal cannula is in patients after extubation who are at low-risk for needing reintubation (Table 2). Hernandez et al. performed a randomized control trial in 527 patients at low-risk for needing reintubation within 72 hours from extubation [13]. Patients were extubated and then randomized to either standard oxygen therapy, which would be considered the standard of care in this group of low-risk patients, or to HFNC. The group extubated to the high-flow nasal cannula had a statistically significant lower rate of reintubation (4.9%) compared to standard oxygen therapy (12.2%). It is unclear why this benefit was seen, but this is certainly a group where many intensivists would not probably attempt the use of high-flow nasal cannula as a therapy as it is usually reserved for more high-risk patients.

**Table 2:** Low Risk for Reintubation Characteristics APACHE II: Acute Physiology and Chronic Health Evaluation II; BMI: body mass index; CHF: congestive heart failure; Table adapted from Hernandez et al. [11]

Low Risk for Reintubation Characteristics
Age < 65
CHF was not an indication for intubation
APACHE II < 12 on the day of extubation
BMI < 30
No airway patency problems
Able to manage secretions
< 2 co-morbidities
Ventilated < 7 days

Pre-oxygenation Prior to Intubation

Intubation of a critically ill patient is a high-risk procedure with high rates of complications, including hypoxemia, hypotension, and even cardiac arrest [14]. The high-flow nasal cannula device has an advantage compared to alternative methods, such as bag-mask ventilation (BMV) and NIPPV. The high-flow nasal cannula device can stay on the patient and provide continued oxygen therapy, as well as possibly provide positive pressure, even during the apneic period compared to BMV and NIPPV, which have to be removed during the intubation procedure. A high-flow nasal cannula may be as effective as NIPPV and superior to standard oxygen therapy for pre-oxygenation prior to intubation in critical patients [14-16].

Do Not Resuscitate (DNR)/Do Not Intubate (DNI) in Respiratory Distress

Peters et al. demonstrated that HFNC may be an effective therapy for patients who are DNI with acute hypoxemia and mild hypercapnia (pCO_2_ < 65) [17]. This therapy was well tolerated and provided acceptable oxygenation without the need for an escalation to NIPPV in 82% of the subjects. This device provides both therapeutic and palliative benefits and may allow for patients to be treated outside of the ICU

Cardiogenic Pulmonary Edema

There isn’t a lot of convincing data to recommend the use of HFNC in patients with cardiogenic pulmonary edema; however, as mentioned earlier, we do know that it can increase intrathoracic pressure and, therefore, is likely to decrease preload. Makdee et al. demonstrated that the high-flow nasal cannula improved the severity of dyspnea in patients with acute cardiogenic pulmonary edema in the emergency department compared to oxygen therapy (NC or NRBM) [18]. More data is needed to demonstrate its effectiveness in this patient population; however, it may be a reasonable therapy for those who are not able to tolerate NIPPV.

Pediatric indications

Bronchiolitis

The majority of pediatric data supporting the use of high-flow nasal cannula outside of neonatal use is in bronchiolitis. Pediatric patients with mild to severe bronchiolitis have the most evidence to support its use. Franklin et al. conducted a multi-centered, randomized controlled trial comparing the use of high-flow nasal cannula (dose = 2 liters per kilogram/min) to standard oxygen in 1,472 infants (< 12 months) with moderate to severe bronchiolitis [19]. Their primary outcome was a failure of treatment requiring an escalation of care. Only 12% or 87/739 infants in the high-flow nasal cannula group failed therapy, while 23% or 167/733 children in the standard oxygen group required an escalation of care. Failure of therapy was defined by having three out of four clinical signs, including persistent tachycardia, tachypnea, and oxygen desaturation, as well as an elevated pediatric early warning score. Interestingly, 61% or 102/167 infants who failed standard oxygen therapy were successfully rescued with the high-flow nasal cannula. No differences were noted in secondary outcomes, including the length of hospital stay, duration of oxygen therapy, pediatric intensive care unit (PICU) admission, or intubation rates.

Other studies have shown the use of HFNC may decrease the need for intubation [20-21], prevent ICU admission [18], and may be as efficacious as NIPPV in the prevention of intubation [21].

Other Uses in Pediatrics

Outside of bronchiolitis, there is limited data to support the use of HFNC in pediatrics. There is some growing evidence for its use in other disease processes where it theoretically may be beneficial. There have been some small retrospective trials examining its benefit with asthma [22-23]. The heated and humidified oxygen may be beneficial to further prevent airway inflammation and bronchospasm. The high-flow rates used with HFNC may also meet the inspiratory demands of the patient, but it may not be as effective in the delivery of aerosolized bronchodilators to distal airways.

Other uses may include pneumonia; however, a lack of data exists here as well and its use would largely be extrapolated from adult use in community-acquired pneumonia. Other disease processes, such as croup, have been retrospectively examined showing that there may exist some benefit [22], as well as in patients in the post-extubation phase after withdrawal of invasive mechanical ventilation [23].

A promising use may be in the transport of critically ill children to larger pediatric hospitals. Newer high-flow devices use battery power and can now be portable to transport children on high-flow devices rather than having to intubate or use NIPPV. Its use appears to be as safe as NIPPV to transport critically ill children between hospitals [24].

## Conclusions

Most of the benefits of the high-flow nasal cannula are from the high-flow rates it can deliver, as well as the heating and humidification of gas. For patients in acute respiratory distress, optimizing your flow rates early will ensure the benefits of the device. Flow rates of 2 liters per/kg/min appear to be safe and effective from pediatric trials up to maximum flow rates of 60 liters/min in adults.

The majority of the benefits, besides heating and humidification, come from the optimal flow. The mnemonic (HIFLOW) will help one to remember the mechanisms of action, including Heated and humidified, meets Inspiratory demands, increases Functional residual capacity (FRC), Lighter, minimizes Oxygen dilution, and Washout of pharyngeal dead space. The most important mechanism may depend on the individual patient's need for the high-flow nasal cannula.

The indications for the use of HFNC in adults include community-acquired pneumonia, post-extubation (even in low-risk patients), pre-oxygenation prior to intubation, DNI patients with respiratory failure, and perhaps in patients with cardiogenic pulmonary edema when NIPPV is not tolerated. In pediatric patients outside the neonatal period, the strongest evidence for the use of the high-flow nasal cannula exists in patients with bronchiolitis, but there is promising evidence that it may benefit other children; however, this data is still lacking.
